# Pleomorphic Adenoma Originating from Heterotopic Salivary Tissue of the Upper Neck: A Diagnostic Pitfall

**DOI:** 10.1155/2017/5767396

**Published:** 2017-11-15

**Authors:** Riccardo La Macchia, Salvatore Stefanelli, Vincent Lenoir, Nicolas Dulguerov, Jean-Claude Pache, Minerva Becker

**Affiliations:** ^1^Division of Radiology, Department of Imaging and Medical Informatics, Geneva University Hospitals, University of Geneva, Geneva, Switzerland; ^2^Division of Head and Neck Surgery, Department of Clinical Neurosciences, Geneva University Hospitals, University of Geneva, Geneva, Switzerland; ^3^Division of Clinical Pathology, Department of Genetic and Laboratory Medicine, Geneva University Hospitals, University of Geneva, Geneva, Switzerland

## Abstract

Pleomorphic adenoma directly arising in the neck is thought to originate from heterotopic salivary gland tissue. In this article, we present the case of a 55-year-old female patient with a histologically proven pleomorphic adenoma located at the left mandibular angle, anteriorly to the sternocleidomastoid muscle and posteroinferiorly to the submandibular gland. As the patient also had an ipsilateral thyroid nodule with coarse calcifications, clinical and radiological features suggested a possible level II metastatic lymph node. However, ultrasound-guided fine needle aspiration cytology and postsurgery histopathological examination revealed a pleomorphic adenoma arising from heterotopic salivary gland tissue unrelated to a benign thyroid nodule. In this article, we provide a review of the existing literature on heterotopic salivary gland tissue and related neoplasms and discuss their imaging presentation.

## 1. Introduction

Heterotopic salivary gland tissue (HSGT) is defined as normal salivary tissue located at sites other than the major and minor salivary glands. Though HSGT has been described in the temporal bone, lymph nodes, and mandible, thyroid, and parathyroid tissues, its presence in the upper and lower neck is very rare, with neoplasms arising from HSGT in the neck being even rarer [1–5]. To the best of our knowledge, only very few cases of ectopic salivary tissue tumors of the upper neck in adults have been reported so far [6]. We report herein the case of a pleomorphic adenoma (benign mixed tumor) arising in the upper neck and mimicking a lymph node metastasis upon clinical examination and cross-sectional imaging. We discuss the imaging features and diagnostic difficulty for this rare entity, providing a radiologic-pathologic correlation.

## 2. Case Report

A 55-year-old female patient, with known sleep apnea and chronic tobacco use, was addressed to the Otorhinolaryngology–Head and Neck Surgery Department of our hospital due to increasing dyspnea upon exertion. Nasofibroscopic exploration revealed leukoplakia on the right vocal cord and bilateral Reinke's edema. On physical examination, a mass located along the anterior border of the left sternocleidomastoid muscle and 2 cm below the left mandibular angle was palpated. The lesion was elastic and not painful, but partly fixed to deeper neck structures.

**Figure 1:**
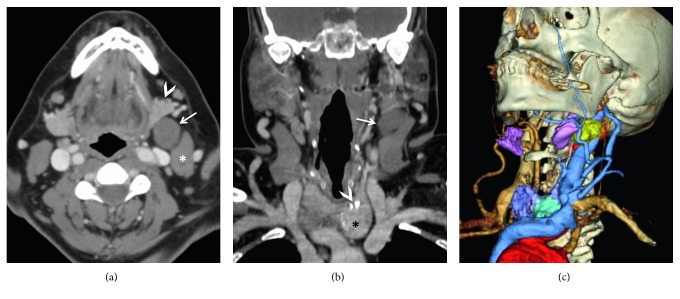
(a) Axial contrast-enhanced CT scan (soft tissue window) showing a well-demarcated mass (*arrow*) located on the left side in the upper cervical lymph node region (group IIA), with adipose cleavage plane between the mass, the submandibular salivary gland (*arrowhead*), and the sternocleidomastoid muscle (*asterisk*). (b) Coronal CT scan reconstruction (soft tissue window) showing a nodular lesion of the left thyroid lobe (*asterisk*) with peripheral macrocalcifications (*arrowhead*). A metastatic lymph node in the ipsilateral level IIA region (*arrow*) was suspected. (c) 3D CT reconstruction showing the left level IIA cervical mass in yellow, the submandibular salivary glands in pink, and the thyroid gland in purple with the left lobe nodule represented in light blue. Arteries are rendered in red and veins in blue.

**Figure 2:**
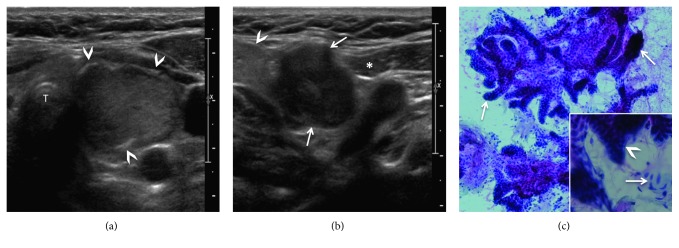
(a) US of thyroid gland (T = trachea) showing the left thyroid nodule, isoechoic to slightly hypoechoic to glandular parenchyma, wide-than-taller shape, hypervascular at color Doppler, with coarse peripheral calcifications (*arrowheads*). No microcalcifications or perinodular thyroid parenchyma invasion is seen. The thyroid nodule was classified TI-RADS 3 (probably benign nodules, <5% risk of malignancy); however, because of its size, FNAC was performed, showing no signs of malignancy. (b) US of the palpable level IIA lesion revealing a hypoechoic, well-delineated, and polylobulated mass (*arrows*) in the left anterior neck triangle located along the anterior border of the sternocleidomastoid muscle (*asterisk*) and submandibular salivary gland (*arrowhead*). (c) FNAC was performed (original magnification, ×25; Papanicolaou (Pap) stain) and revealed tumor epithelial part (*arrows*) with squamoid epithelial cells (*arrowhead*, inset in (c), original magnification, ×100; Pap stain) and myoepithelial cells (*arrow*, inset in (c)), thus suggesting the diagnosis of pleomorphic adenoma.

To further assess this lesion, a contrast-enhanced computed tomography (CT) of the neck was performed. It revealed a 23 × 17 × 25 mm solid nodule with coarse calcifications located in the left thyroid gland and an ipsilateral 24 × 25 × 35 mm well-delineated hypodense mass located in the level IIA lymph node group (Figure 1). Although the CT features were not typical of a metastatic lymph node, due to the presence of an ipsilateral suspicious thyroid nodule, a ipsilateral lymph node metastasis was suspected. Ultrasonography (US) with subsequent US-guided fine needle aspiration cytology (FNAC) was carried out. The thyroid lesion was slightly hyperechoic to the muscle, hypervascular on color Doppler, with coarse peripheral calcifications, whereas the level IIA lesion was well delineated and solid, being hypoechoic to muscle tissue, homogenous, and hypovascular (Figure 2). The level IIA mass was separated from the submandibular gland by the facial vein, and a fatty cleavage plane was clearly visible between the mass and submandibular gland, both upon CT and US imaging. FNAC of the thyroid nodule showed follicular cell clusters organized into macrofollicles, compatible with a benign thyroid nodule. FNAC of the level IIA mass showed clusters of benign epithelial cells, myxoid substance, and myoepithelial cells, suggesting the diagnosis of pleomorphic adenoma.

**Figure 3:**
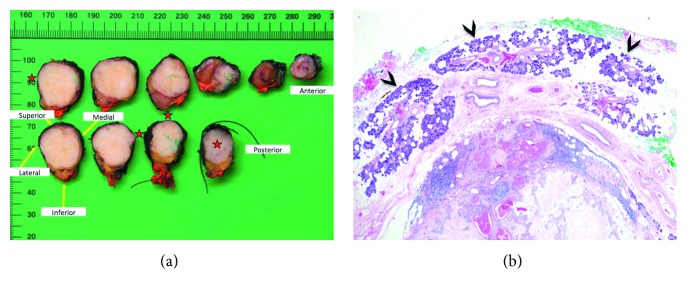
(a) Macroscopic aspect of the level IIA mass after surgical excision: well-delineated white solid lesion with tumor margin smaller than 0.1 cm (*red stars*). (b) Histopathology (original magnification, ×25; HE stain) revealing that the well-delineated tumor (lower part of the picture) is surrounded by a normal serous salivary gland (*arrowheads*), thus suggesting the diagnosis of pleomorphic adenoma of an ectopic minor salivary gland.

The patient underwent surgery under general anesthesia, and histopathological examination confirmed the FNAC findings. Histopathology indicated the level IIA mass to be an encapsulated classic pleomorphic adenoma with a predominant myoepithelial component, surrounded by clusters of normal salivary gland tissue embedded in fatty tissue (Figure 3). Three reactive lymph nodes were found in the tumor vicinity but without any evidence of malignant cells or perineural spreading. The patient's postoperative recovery proved uneventful, and she was discharged 2 days later. No lesion recurrence was found at the 2-year follow-up visit.

## 3. Discussion

At the head and neck level, salivary tissue is present in the major salivary glands (parotid, submandibular, and sublingual glands), minor salivary glands (upper aerodigestive tract), and accessory salivary glands (immediate anterior periparotid region), and this is present in association with branchial cleft anomalies, or more rarely, as heterotopic salivary gland tissue (HSGT). HSGT is defined as salivary tissue outside the major, minor, and accessory salivary glands, with no clinical or histological features of branchial cleft anomalies [7]. Though rare, HSGT in the head and neck can be seen in a variety of locations, such as lymph nodes, external auditory canal, mandible, mastoid bone, middle ear, tongue, anterior sternocleidomastoid muscle and sternoclavicular joint, thyroid and parathyroid glands, and upper and lower neck regions [2]. The embryological basis for this rare condition is unknown. One of the theories assumes HSGT in the neck to be caused by defective closure of the precervical His sinus, with internal heteroplasia [3, 8].

HSGT is susceptible to the same disorders than those affecting the major and minor salivary glands, namely, infectious or inflammatory disorders, as well as neoplastic diseases. Both benign and malignant neoplasms can occur in HSGT. The literature suggests that pleomorphic adenoma is the second most common neoplasm arising from HSGT after Warthin's tumor [2]. Although pleomorphic adenoma arising from HSGT is rare, several authors have suggested that it should be systematically considered in the differential diagnosis of neck swellings.

Most pleomorphic adenomas arise in the parotid glands, in which case the diagnosis is usually straightforward upon imaging. Pleomorphic adenomas have mainly well-defined borders and most often display a myxoid matrix on histopathology examination, with typically high signal intensity on T2-weighted sequences, low signal intensity on T1-weighted sequences, and moderate contrast enhancement. On diffusion-weighted sequences, there is no restricted diffusivity. On CT, the tumors display only minimal enhancement; on US, they are strongly hypoechoic, while being hardly vascularized on Doppler-US. The combination of typical location in the parotid gland, well-defined borders, and abovementioned imaging features enables a correct presumptive diagnosis in most cases. Nevertheless, in the presence of atypical features (poorly defined borders and hypercellular variants with lower signal intensity on T2-weighted sequences) or atypical location, US-guided FNAC is recommended prior to surgery. In the current case, the presence of a thyroid nodule with coarse calcifications, along with a hypoechoic mass on US located in the ipsilateral level II lymph node group, suggested a possible malignant tumor with lymph node metastasis. In this case, the difficulty in establishing the correct diagnosis arose from the unusual location of the pleomorphic adenoma, as well as from its association with a thyroid nodule.

In this case, US-guided FNAC yielded the correct diagnosis. US, whether with or without FNAC, is generally recommended as the first preoperative radiological examination for any mass of the head and neck regions [9]. Despite the rarity of salivary lesions, most nonneoplastic lesions as well as benign and malignant tumors are easily recognizable and properly diagnosed based on cytological specimens [10]. The diagnostic value of FNAC in the assessment of salivary gland lesions has been carefully evaluated and reported in the literature, with a consensus among authors regarding its high specificity and diagnostic precision [10]. The accuracy of FNAC depends on the pathologist's experience and precision [11]. This little-invasive method allows for an excellent distinction to be made between benign and malignant tumors of the salivary glands, while providing other relevant advantages like low cost and ease of execution and accessibility, in addition to being relatively painless, well tolerated, and associated with a very low risk of cancer cell implantation along the needle pathway [11]. However, MRI is the preferred imaging modality designed to evaluate neoplastic lesions of salivary glands, especially in the case of larger and aggressive tumors. MRI also proves useful in differentiating tumor types, particularly in the diagnosis of pleomorphic adenoma (discussed earlier) [9].

Though the FNAC samples of pleomorphic adenoma of any location are often highly reliable, they prove to be nondiagnostic in the event of complex tumor composition [9]. It is well known and accepted that inconclusive salivary gland FNACs, with significant impact on clinical and surgical management, are due to several factors, such as heterogeneous morphology, resemblance to normal salivary tissue, cystic components, clear cells, and oncocytic metaplasia [10]. In salivary gland FNAC specimens, diagnostic difficulties with FNAC are most commonly encountered for the differentiation between pleomorphic adenoma and some subtypes of adenoid cystic carcinomas, basal cell adenomas, low-grade mucoepidermoid carcinomas, and acinic cell carcinomas. The diagnosis proves challenging in low-grade mucoepidermoid carcinomas, with a thick mucinous fluid background and paucicellular smear. In such cases, malignant tumors are mistakenly reported as benign lesions [11].

Although pleomorphic adenoma is associated with good prognosis, the treatment of choice is surgical excision with free margins in order to prevent local recurrence and malignant transformation into carcinoma ex pleomorphic adenoma (CEPA), carcinosarcoma (true malignant mixed tumor) [12], or metastasizing pleomorphic adenoma [13]. According to the literature, the risk of malignant transformation is 3–15%, increasing with the time elapsed prior to diagnosis and tumor size [14]. CEPA is both an aggressive tumor and feared complication of long-standing pleomorphic adenoma, accounting for 11.6% of all malignant salivary gland tumors [14]. A few cases of CEPA originating from minor salivary gland tissue in the neck region have been described, yet with only one case originating from HSGT available in the English literature [14].

Multifocal local recurrence of pleomorphic adenomas, a typical feature, is well described in the literature, probably accounted for by close margins, pseudocapsular tears, or tumor spillage upon surgical resection. Hypocellular tumors, younger age at initial presentation, female gender, and tumor recurrence are considered critical factors for an increased risk of recurrence [15]. The recurrence probability is higher within the first 10 years following initial surgery, and, in rare instances, recurrences have been reported occurring as long as 45 years following initial surgery [16, 17]. Regular US follow-up over the patient's lifetime is recommended for superficial head and neck pleomorphic adenomas. MRI follow-up is considered useful in selected cases, nonaccessible to US [18].

## 4. Conclusion

Ectopic pleomorphic adenoma is one of the differential diagnoses to be taken into account when facing any level II cervical mass with well-defined borders at imaging, hypoechoic and hypovascular US features, and with minor but homogeneous contrast enhancement at CT. As FNAC is a fairly accurate preoperative procedure for the diagnosis of pleomorphic adenoma, it should be carried out under US guidance to confirm the diagnosis.

## Abbreviations

CT: Computed tomography
CEPA: Carcinoma ex pleomorphic adenoma
FNAC: Fine needle aspiration cytology
HSGT: Heterotopic salivary gland tissue
US: Ultrasonography.
